# Encephalomyocarditis virus infection in an Italian zoo

**DOI:** 10.1186/1743-422X-7-64

**Published:** 2010-03-18

**Authors:** Elena Canelli, Andrea Luppi, Antonio Lavazza, Davide Lelli, Enrica Sozzi, Ana M Moreno Martin, Daniela Gelmetti, Ernesto Pascotto, Camillo Sandri, William Magnone, Paolo Cordioli

**Affiliations:** 1 Istituto Zooprofilattico Sperimentale della Lombardia e dell'Emilia Romagna "B Ubertini" (IZSLER), Via Bianchi, 7/9 - 25124 Brescia, Italy; 2 Natura Viva Zoo, Loc Figara, 40 - 37012 Bussolengo, Verona, Italy; 3 Department of Animal Science, Udine University, Via delle Scienze, 208 - 33100 Udine, Italy

## Abstract

A fatal Encephalomyocarditis virus (EMCV) infection epidemic involving fifteen primates occurred between October 2006 and February 2007 at the *Natura Viva Zoo*. This large open-field zoo park located near Lake Garda in Northern Italy hosts one thousand animals belonging to one hundred and fifty different species, including various lemur species. This lemur collection is the most relevant and rich in Italy. A second outbreak between September and November 2008 involved three lemurs. In all cases, the clinical signs were sudden deaths generally without any evident symptoms or only with mild unspecific clinical signs. Gross pathologic changes were characterized by myocarditis (diffuse or focal pallor of the myocardium), pulmonary congestion, emphysema, oedema and thoracic fluid. The EMCV was isolated and recognized as the causative agent of both outbreaks. The first outbreak in particular was associated with a rodent plague, confirming that rats are an important risk factor for the occurrence of the EMCV infection.

## Background

Encephalomyocarditis virus (EMCV) is a single stranded *Cardiovirus *belonging to the *Picornaviridae *family. It is spread worldwide and it is recognized as a pathogen found mainly in pigs but also in non-human primates and in a variety of domestic, captive, non-domestic and wild animals. Several outbreaks of fatal EMCV infections have been described in zoos in Australia and the USA [1-5]. Rodents and in particular rats and mice are usually considered the natural host and reservoir of this virus. They are suspected of contaminating feed or water, through which the infection spreads to susceptible animals.

EMCV can cross the species barrier, as demonstrated in some zoo outbreaks involving multiple animal species [4,5]. Recently, the interest in this virus has increased because of possible pig-to-human transmission by xeno-transplantation. Until today, human cases have been fortunately very rare [6] and although the infection is possible the risk appears to be almost negligible.

This report describes an EMCV infection occurred in non-human primates housed in an Italian zoo. Specifically two outbreaks are described from a clinical, anatomopathological and diagnostic point of view.

## Case presentation

### Outbreaks

The *Parco Natura Viva *zoo in Bussolengo (Verona, Italy) houses one thousand animals belonging to one hundred and fifty different species including various primates and it hosts the most relevant captive lemur population in Italy. The first outbreak occurred between October 2006 and February 2007, when fifteen primates out of a total of ninety-six (15.6% morbidity) and belonging to six different species died: one black lemur (*Eulemur macaco macaco*), three ring-tailed lemurs (*Lemur catta*), three red-ruffed lemurs (*Varecia variegata rubra*), two white-fronted lemurs (*Eulemur albifrons*), four barbary macaques (*Macaca sylvanus*) and two common marmosets *(Callithrix jacchus)*. All lemurs (forty-two animals) were housed in big hutches, one for each species, all situated in the same area of the Park; the common marmosets (two animals) were housed in a hutch within a greenhouse, while the twenty-five barbary macaques (*Macaca sylvanus*) were housed in a separated hutch on small island located in front of the greenhouse and about 200 meters far from the lemurs. The fifteen primates mentioned before died without any clear apparent predictive symptoms or only with mild unspecific clinical signs. Indeed, the clinical course was very rapid in most cases, which were described by the zoo's veterinarian as sudden asymptomatic deaths. There were only certain cases, which started with lethargy, decreased activity, weakness and malaise, which then caused death after 12-24 hours.

A second outbreak occurred between September and November 2008, involving three red-ruffed lemurs (*Varecia variegata rubra*). More details on the animals involved during the two outbreaks are shown in table 1.

**Table 1:** Clinical and pathological findings during the two outbreaks.

Species	Sex	Death date	Clinical signs	Gross pathology	EMCV investigation results
*Lemur catta*	n.r. °	15-10-06	Sudden death	No gross lesions were observed	Pos

*Lemur catta*	m	20-10-06	Lack of coordination	Pericardic haemorrhages, pulmonary emphysema and oedema, meningeal congestion	Pos

*Lemur catta*	m	4-11-06	Sudden death	Sero-haemorrhagic thoracic fluid, pulmonary oedema, catharral enteritis, pericardic haemorrhages, cardiomegaly, whitish necrotic foci, pulmonary emphysema, ascites, abdominal organs congestion, mild liver hyperaemia, meningeal congestion	Pos

*Eulemur macaco macaco*	m	17-11-06	Anorexia, sensory depression	Very severe pulmonary oedema	Pos

*Eulemur albifrons*	m	27-11-06	Sudden death	Diffuse pulmonary edema, sero-haemorrhagic thoracic fluid, pericardic haemorrhages, pulmonary emphysema, meningeal congestion, catharral enteritis	Pos

*Eulemur albifrons*	m	16-12-06	Lethargy, sensory depression	Thoracic fluid, severe pulmonary edema, cardiomegaly, presence of nematodes (*Trichuris *spp.) in the stomach, sero-haemorrhagic thoracic fluid, pericardic haemorrhages, pulmonary emphysema, meningeal congestion	Pos

*Macaca sylvanus*	m	27-12-06	Sudden death	Mild pulmonary oedema	Pos

*Varecia variegata rubra*	n.r.	27-12-06	Sudden death	Mild pulmonary oedema, evident cardiomegaly and grey-white necrotic foci of the myocardium	Pos

*Macaca sylvanus*	n.r.	30-12-06	Sudden death	Necrosis of the posterior fingers and partial necrosis of anterior fingers, marginal lobular pulmonary haemorrhages, evident cardiomegaly, abundant hydropericardium	Neg

*Macaca sylvanus*	n.r.	05-01-07	Sudden death	No gross lesions were observed	Pos

*Callithrix jacchus*	m	16-01-07	Sudden death	Lymphomegaly, kidney pallor, epathomegaly, cardiomegaly and grey-white necrotic foci of the myocardium, pulmonary oedema, ascite	Pos

*Callithrix jacchus*	f	19-01-07	Sudden death	Mild pulmonary oedema	Neg

*Varecia variegata rubra*	f	21-01-07	Sudden death	Thoracic fluid, evident pulmonary oedema, cardiomegaly and grey-white necrotic foci of the myocardium	Pos

*Varecia variegata rubra*	n.r.	02-02-07	Sudden death	Mild pulmonary oedema and grey-white necrotic foci of the myocardium	Pos

*Macaca sylvanus*	n.r.	07-02-07	Sudden death	Catharral enteritis, presence of nematodes (*Strongiloides *spp.) in the small intestine	Pos

*Varecia variegata rubra*	m	23-09-08	Sudden death	Pulmonary oedema and grey-white necrotic foci of the myocardium	Pos

*Varecia variegata rubra*	m	04-11-08	Sensory depression	Pulmonary oedema and grey-white necrotic foci of the myocardium	Pos

*Varecia variegata rubra*	f	06-11-08	Sudden death	Pulmonary oedema and grey-white necrotic foci of the myocardium	pos

°n.r. = not reported

Animals belonging to other species were not affected by these symptoms neither was abnormal mortality detected.

### Epidemiological and Diagnostic investigations

The epidemiological investigation was done in order to find out the causes of the introduction and the spreading of EMCV in the Park.

In all cases, necropsy was performed and selected internal organs were sampled and submitted for parasitological, bacteriological, virological, histopathological and toxicological examinations.

The copro-parasitological analysis was made on intestinal content using standard qualitative methods (sedimentation and floatation). Bacteriological exams were performed on lungs, small and gross intestines, kidneys, liver, brain and spleen following a standardized protocol. Toxicological examination was focused on detecting rodenticidals in liver and gastric content samples.

For the virological examinations lungs, spleen, brain and heart were homogenized in minimal essential medium (MEM) (1 g/10 ml) containing antibiotics and clarified by centrifugation. The supernatants of organ homogenates were separately inoculated on VERO (African green monkey kidney cells) and BHK21 (baby hamster kidney) cells. The presence and identification of EMCV particles in cell culture lysates was found using both a MAbs-based sandwich ELISA produced by IZSLER [7] and a negative staining immuno-electron-microscopy using the Airfuge method and employing a positive reference serum produced by IZSLER (Novara 86 strain). The grids were stained with 2% NaPT, pH 6,8 and examined with a TEM Philips CM10 operating at 80 kV.

For the histopathology, portions of myocardium, lungs, small and large intestine, kidney, liver, pancreas, spleen and brain were fixed in 10% buffered formalin and 5 μm-thick paraffin-embedded sections were obtained and stained with haematoxylin-eosin. Immunohistochemistry was performed only on samples those showed histological lesions, using the monoclonal antibody (MAb) 3E5 produced by IZSLER laboratories specific for EMCV in a biotin-streptavidin staining method.

Nine sera, some previously collected by zoo veterinarians for routinely laboratory investigations and others from different lemurs survived to the outbreaks, were tested also for EMCV antibodies using a competitive ELISA [7]. Specifically, these sera were collected from nine different animals: before the outbreaks on 28/01/06 (n° 1 serum) and 15/02/06 (n° 1); after the first outbreak on 18/02/2008 (n° 1), 08/2008 (n° 2) and 30/10/2008 (n° 1) and after the second outbreak on 01/2009 (n° 2) and 15/02/2009 (n° 1). In order to avoid unnecessary stress to animals it was decided to limit the serological investigation for EMCV antibodies only to these sera.

After identifying the EMCV (see below) and considering that rodents are an important risk factor for EMCV infection, a rodent control was made for all areas used for animal food storage and preparation to avoid any possible contact between rodent feces and food. Ten rats were also captured in the immediate area of the lemur enclosure and immediately sacrificed. It was impossible to take blood for serological investigations. Samples of brain, liver, spleen, heart, intestine and urine were tested separately using cell cultures (VERO and BHK21).

## Results and Discussion

The epidemiological investigation showed two important findings. The first one was the increased number of rats in the Park. For this reason, a supplementary rat control program was in progress during the first outbreak, although a rat disinfection program was normally applied in the Zoo. The second point was relative to the 15 primates involved in the outbreak. As a matter of fact they was fed with the same food and attended by the same zookeeper. These results could suggest a possible EMCV introduction and spreading directly by rats and indirectly by food and fomites contaminated by rat urine and faeces.

**Figure 1:**
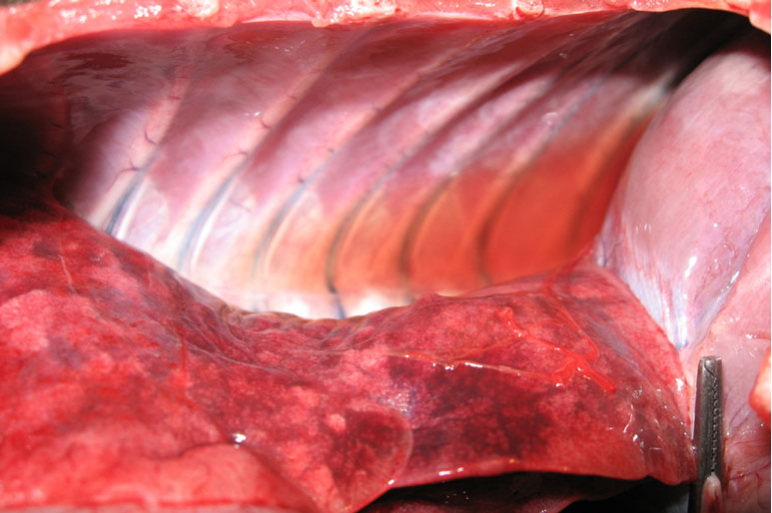
**Thoracic cavity of a *Callithrix jacchus *that died during the first outbreak**. Sero-hemorragic transudate

**Figure 2:**
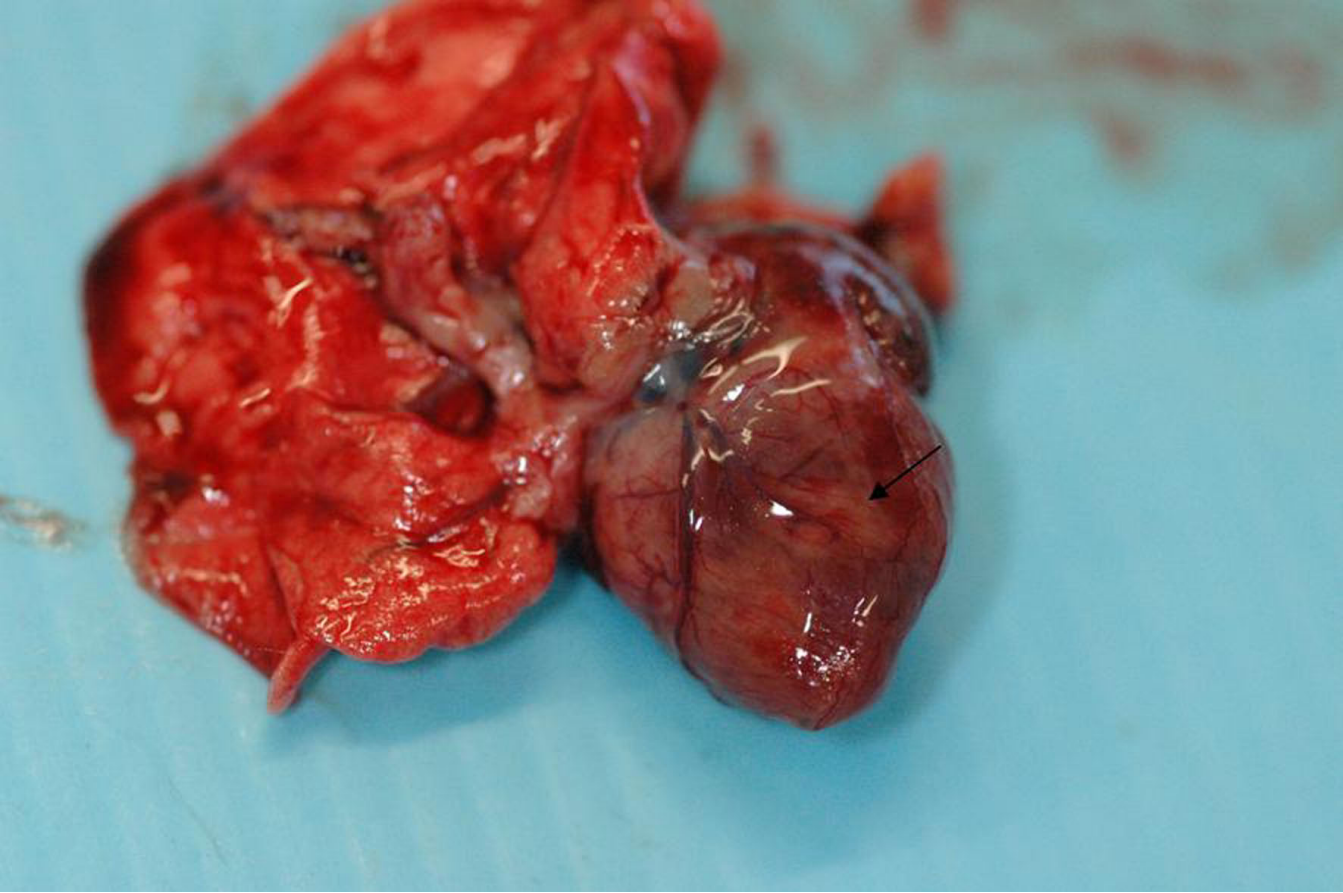
**Heart of a *Lemur catta *that died during the first outbreak**. Some typical white foci of necrosis in the myocardium

At necropsy, a small amount of fluid transudate was observed in both thoracic (Figure 1) and abdominal cavities (hydrothorax and ascites), but the main lesions were primarily limited to the cardiovascular system. Hydropericardium was associated with pericardic hemorrhages, mild cardiomegaly, grey-white necrotic foci of the myocardium and petechiae or ecchymoses on the epicardial surface (Figure 2). Lungs were involved in most cases showing mild to severe pulmonary emphysema, moderate oedema and congestion with blood-tinged foam in the trachea. In some cases, the enteric tract had a reinforced parietal and mesenteric vascular plot. In one animal, brain lesions (hyperemia and oedema) and meningeal congestion were also observed.

During necropsy, some parasites were found in the stomach of a *Eulemur albifrons *and in the intestine of a *Macaca sylvanus*. The identification of adults and eggs was based on the morphology and micrometric study, showing the presence of *Trichuris *spp. and *Strongiloides *spp. respectively.

Bacteriological and toxicological investigations were negative.

**Figure 3:**
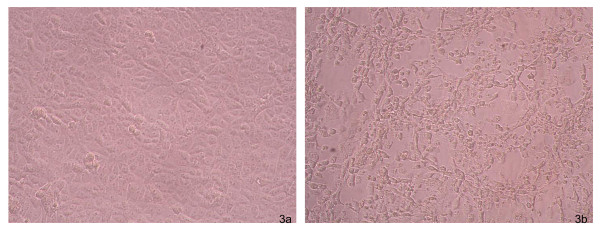
**Vero cells culture**. a) Negative control: uninfected cell monolayer. (20× magnification). b) Evident cytopathic effect due to EMCV, 72 hours after the infection. (20× magnification).

After 48-72 h post-inoculation of homogenates of different organs, VERO and BHK21 cell cultures showed a cytopathic effect (CPE) (Figure 3) and the virus was identified as EMCV by using the MAbs-based sandwich ELISA. Virus isolation was obtained from all sampled organs.

**Figure 4:**
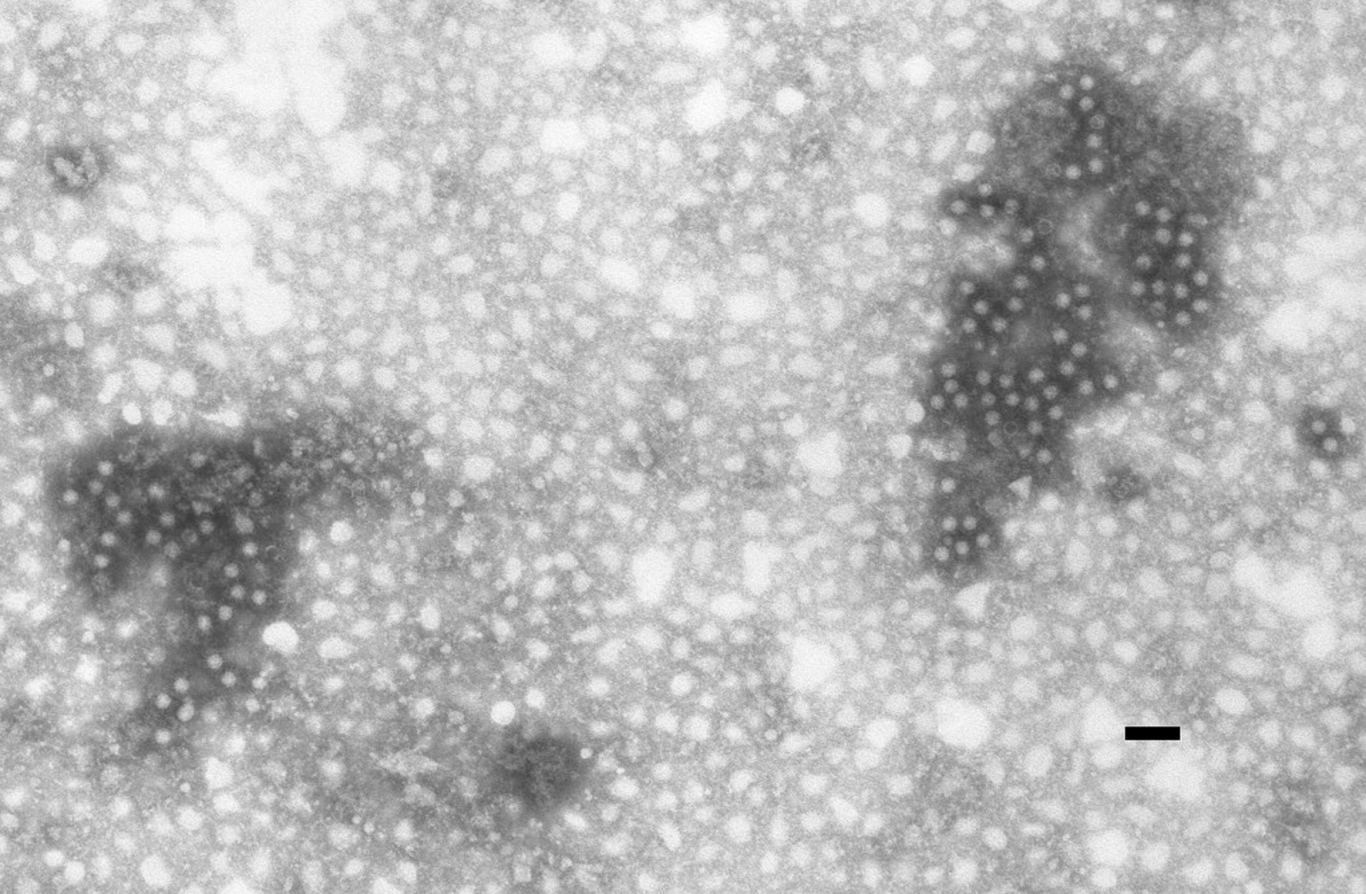
**Electromicrograph**. EMCV particles using IEM. (NaPT 2%. Bar = 100 nm).

**Figure 5:**
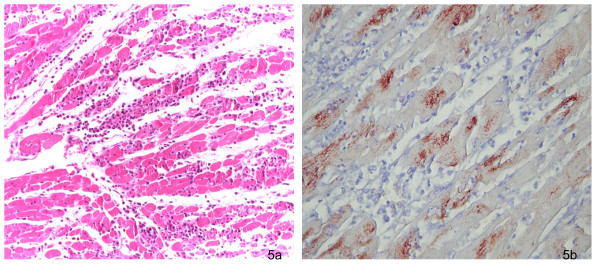
**Histological examination of myocardium**. a) Severe myocarditis characterized by an interstitial infiltration of lymphocytes and neutrophilic granulocytes. (Hematoxylin-eosin, 40× magnification) b) EMCV - immunopositive myocardiocytes using immunohistochemistry. (100× magnification).

Furthermore the IEM examination was used to confirm this diagnosis (Figure 4). Although EMCV isolation was obtained from lungs, spleen, brain and heart, histological lesions were generally confined to the cardiovascular system (Figure 5a). The myocardium had hydropic degeneration with focal areas of necrosis and different degrees of lymphocytes and neutrofilic granulocytes interstitial infiltrations. Diffuse linfangectasia and peri-vasal hemorrhages associated with mixed necrotizing vasculitis, multifocal inflammatory infiltrations and necrotizing phenomena were also evident. Multifocal areas of colliquative miocardic necrosis were found, associated with prevalent granulocytic infiltration. These lesions were often situated at the basis of papillary muscles and at the inter-ventricular sect level. An oedema was evident in the peripheral areas of the lesions, while compensative hypertrophy was present in the atrium. The histological examination of the small and large intestines, kidneys, liver and spleen did not show any specific lesions. These findings partially agree with the results of an experimental infection with EMCV in several primates (*Cynomogolus macaques*) reported by La Rue *et al*., 2003 [8]. This work revealed the presence of EMCV RNA using rt-PCR in the blood, spleen, liver, heart, kidney, brain and skeletal muscles after the EMCV inoculation in four *Cynomogolus macaques*. This viral RNA localization was associated with several pathological changes only in the heart and the brain.

The encephalic analysis of primates which died during the two outbreaks, showed a clear congestion with moderate meningeal perivascular hemorrhages, clear neuronal satellitosis with neuronal degenerative changes and areas of neuronal necrosis only in one case. The immunohistochemistry was performed on the brain and on the myocardium. No immunopositive signal was apparent in the brain, while EMCV immunopositive myocardiocytes were observed in all cases and the intensity and distribution of the immuno-labelling, agreed with the severity of the histological lesions (Figure 5b). These results could be explained considering that Encephalomyocarditis virus is principally cardiotropic in non human primates [8]. Probably the histological lesions observed in the SNC were not directly linked to EMCV local replication but they could be due to anoxia with brain damage, consequent to heart attack or arhytmia. Indeed, when EMCV replicates in the SNC of pig and non human primates, the lesions are generally represented by foci of perivascular cuffing and lymphocytic infiltration in the cerebral cortex, meninges and cerebrum [8]. The main clinical, pathological and diagnostic findings are reported in Table 1.

The ELISA test on the collected sera was negative. As described before the Zoological Park hosts many different species. A large number of them are sensible to the EMCV infection [1-5,9-13]. In agreement with the Direction of the Park, it was decided to avoid a random collection of blood samples in captive wild animals in order to prevent useless stress in the animals. Therefore, it was not possible to show if other species were infected or not. Nevertheless, no clinical signs or sudden deaths were observed in other species than primates.

The sampled rats were all negative to laboratory investigation and in particular to viral isolation. Nevertheless similar outcomes were described in literature by other authors [4] and a successful isolation, even if at low rate, was demonstrated after an experimental infection by examining lymphoid tissues [14].

## Conclusions

This report describes two outbreaks of ECMV which occurred in zoo captive primates in Italy, confirming the risk that this virus has present to such species and their high susceptibility to the infection. Furthermore, EMCV could be a potential issue for other zoological park animals as it may involve several other species. EMCV infections may often be clinically non-evident and only in certain cases they show extreme virulence, lethality and severity [1,4,5,9]. Thus, EMCV should always be included in differential diagnosis when sudden death of primates without obvious symptoms occurs, in particular when there is myocarditis at necropsy.

The time period of the two cases confirmed the seasonality of the infection, typically reported during cold months [10].

Rats are considered the source of the virus since they can excrete it for long periods in feces and urine, contaminating food and water. Furthermore, their carcasses can be infectious through ingestion [10]. The first outbreak was associated with a rodent plague and, even if no captured rats were positive to the virus isolation, an important epidemiological connection between rats and this outbreak could be pointed out. The outbreak started when the number of rats increased in the zoo and was completely solved only when a rodent control program, feeding hygienic practices and rigid bio-security measures were applied in the zoo. This agrees with the various studies in which mice and rats were associated with clinical outbreaks of EMCV, not only in wild animals [1,3-5,9-13,15-17], but often also in pigs herds [18-20].

No information is available on the possibility of introducing EMCV by another species residing in the zoo. However, neither other rodents were observed during the two outbreaks nor new animals were introduced in the zoo in connection with the EMCV clinical onset.

The reoccurrence of a second outbreak after some months is indeed difficult to explain. In addition to the role of rats as carriers, the possibility of reactivation of EMCV in persistently infected animals should not be completely excluded [21].

All tested lemur sera were negative. Unfortunately, to avoid dangerous stress to the animals, it is not currently possible to collect further samples to verify the serological status of the surviving animals or to check their potential status of persistently infected. Further investigation will be necessary to assess whether, as previously suggested [4], immunity is not protective against later exposure to this virus. In any case, due to the environmental resistance of the virus, the recrudescence of the infection may be an important proof of this hypothesis.

Bearing in mind that human cases are few and rare, but also considering the assumption of a zoonotic nature of ECMV, these finding are nevertheless of public concern.

## Competing interests

The authors declare that they have no competing interests.

## Authors' contributions

EC performed virological and serological analysis, set the results' interpretation and drafted the manuscript. ALu conducted epidemiological investigation, performed some necropsies and was a major contributor in writing the manuscript and interpreting the data. ALa performed the immuno-electron microscopy exams and helped in the writing and in the critical revision of the manuscript and ensured the overall supervision. DL, ES, AMMM were involved in the execution of virological analysis and in the interpretation of the analytical data. DG and EP carried out the histopathology and immuno-istochemistry exams and their interpretation. EP also performed necropsies and made the interpretation of clinical and anatomo-pathological outcomes. CS, WM collected the patient samples, performed all the clinical exams and the majority of the necropsies, gave all the data about the zoo animals involved and participated in the epidemiological investigations. PC conceived the study, made the interpretation of data and all the analysis were carried out under his supervision and coordination. All authors read and approved the final manuscript.

## References

[B1] Gaskin JM Jorge MA Simpson CF Lewis AL Olson JH Schobert EE Wollenman EP Marlowe C Curtis MM The tragedy of encephalomyocarditis virus infection in zoological parks of Florida Proceedings American Association of Zoo Veterinarians 1980 1 7

[B2] Gaskin JM Andresen TL Olsen JH Schobert EE Buesse D Lynch JD Walsh M Citino S Murphy D Encephalomyocarditis in zoo animals: Recent experiences with the disease and vaccination Proceedings of the 1st International Conference on Zoological and Avian Medicine 1987 491

[B3] McLelland D Kirkpatrick JF Rose K Dixon R Studies on encephalomyocarditis virus (EMCV) in a zoologic context AAZV, AAWV, ARAV, NAZWV Joint Conference 2001 337

[B4] Reddacliff L Kirkland PD Hartley WJ Reece RL Encephalomyocarditis virus infections in an Australian zoo J Zoo Wild Med 1997 28 2 153 157 9279403

[B5] Wells SK Gutter AE Soike KF Baskin GB Encephalomyocarditis virus: Epizootic in a zoological collection J Zoo Wildl Med 1989 20 291

[B6] Oberste MS Gotuzzo E Blair P Nix WA Ksiazek TG Comer JA Rollin P Goldsmith CS Olson J Kochel TJ Human Febrile Illness Caused by Encephalomyocarditis Virus Infection Peru Emerg Infect Dis 2009 15 4 640 646 10.3201/eid1504.081428 PMC2671410 19331761

[B7] Brocchi E Carra E De Simone F Development of monoclonal antibodies based ELISAs for the detection of Encephalomyocarditis viruses (EMCV) and of EMCV-induced antibodies Proceedings of the S.I.Di.L.V.,13 nov 1998, Salsomaggiore (PR) 1998 31

[B8] La Rue R Myers S Brewer L Shaw DP Brown C Seal BS Njenga MK A wild-type Porcine Encephalomyocarditis virus containing a short poly(C) tract is pathogenic to mice, pigs and Cynomolgus macaques Journal of Virology 2003 77 9136 9146 10.1128/JVI.77.17.9136-9146.2003 12915530 PMC187386

[B9] Citino SB Gaskin HJH Wickham DJ Fatal Encephalomyocarditis virus infection in Sumatran orangutan (*Pongo pygmaeus abelii*) J Zoo Wildl Med 1998 19 214 218

[B10] Murname TG Steele JH Encephalomyocarditis CRC Handbook Series in Zoonoses, Section B: Viral Zoonoses 1981 The Iowa State University Press Ames, Iowa 137 147

[B11] Billinis C Encephalomyocarditis virus infection in wildlife species in Greece J Wildl Dis 2009 45 2 522 6 19395765 10.7589/0090-3558-45.2.522

[B12] Gutter AE Fowler ME Encephalomyocarditis in zoo animals Zoo and Wild Animal Medicine 1993 2 W.B. Saunders Co, Philadelphia 50 51

[B13] Hubbard GB Soike KF Butler TM An Encephalomyocarditis virus epizootic in a baboon colony Lab Anim Sci 1992 42 233 239 1320151

[B14] Spyrou V Maurice H Billinis C Papanastassopoulou M Psalla D Nielen M Koenen F Papadopoulos O Transmission and pathogenicity of encephalomyocarditis virus (EMCV) among rats Vet Res 2004 35 1 113 122 10.1051/vetres:2003044 15099508

[B15] Grobler DG Raath JP Braack LEO Keet DF Gerdes GH Barnard BJH Krick NPJ Jardine J Swanepoet R An outbreak of Encephalomyocarditis-virus infection in free ranging African elephants in the Kruger National Park Onderstepoort J Vet Res 1995 62 97 108 8600443

[B16] Seaman JT Finnie EP Acute myocarditis in a captive African elephant (*Loxodonta africana*) J Wild Dis 1987 23 170 171 10.7589/0090-3558-23.1.170 3820423

[B17] Simpson CF Lewis AL Gaskin JM Encephalomyocarditis virus infection of captive elephants J Am Vet Med Assoc 1977 171 902 905 200596

[B18] An DJ Jeong W Jeoung HY Yoon SH Kim HJ Choi CU Park B Encephalomyocarditis in Korea: serological survey in pigs and phylogenetic analysis of two historical isolates Vet Microbiol 2009 28 37 4 10.1016/j.vetmic.2009.01.005 19200668

[B19] Bakkali Kassimi L Madec F Guy M Boutrouille A Rose N Cruciere C Serological survey of Encephalomyocarditis virus infection in pigs in France Vet Rec 2006 14,159 16 511 4 10.1136/vr.159.16.511 17041064

[B20] Maurice H Nielen M Brocchi E Nowotny N Kassimi LB Billinis C Loukaides P O'Hara RS Koenen F The occurrence of encephalomyocarditis virus (EMCV) in European pigs from 1990 to 2001 Epidemiol Infect 2005 133 547 5 10.1017/S0950268804003668 15962562 PMC2870279

[B21] Billinis C Paschaleri-Papadopoulou E Psychas V Vlemmas J Leontides S Koumbati M Kyriakis CS Papadopoulos O Persistence of encephalomyocarditis virus infection in Vet Microbiol 1999 70 171 177 10.1016/S0378-1135(99)00137-6 10596801

